# Substrate Stiffness Influences Doxorubicin-Induced p53 Activation via ROCK2 Expression

**DOI:** 10.1155/2017/5158961

**Published:** 2017-01-16

**Authors:** Takahiro Ebata, Yasumasa Mitsui, Wataru Sugimoto, Miho Maeda, Keigo Araki, Hiroaki Machiyama, Ichiro Harada, Yasuhiro Sawada, Hideaki Fujita, Hiroaki Hirata, Keiko Kawauchi

**Affiliations:** ^1^Frontiers of Innovative Research in Science and Technology, Konan University, 7-1-20 Minatojima-Minamimachi, Chuo-ku, Kobe, Hyogo 650-0047, Japan; ^2^Department of Bioscience, School of Science and Technology, Kwansei Gakuin University, 2-1 Gakuen, Sanda, Hyogo 669-1337, Japan; ^3^Laboratory for Comprehensive Bioimaging, Riken Qbic, 6-2-3 Furuedai, Suita, Osaka 565-0874, Japan; ^4^WPI, Immunology Frontier Research Center, Osaka University, 1-3 Yamadaoka, Suita, Osaka 565-0871, Japan; ^5^Laboratory for Mechanical Medicine, Nadogaya Research Institute, Nadogaya Hospital, 687-4 Kashiwa, Chiba 277-0032, Japan; ^6^Department of Rehabilitation for the Movement Functions, Research Institute, National Rehabilitation Center for Persons with Disabilities 4-1 Namiki, Tokorozawa, Saitama 359-8555, Japan; ^7^Nagoya University Graduate School of Medicine, 65 Tsurumai, Showa-ku, Nagoya, Aichi 466-8550, Japan; ^8^Department of Molecular Oncology, Institute for Advanced Medical Sciences, Nippon Medical School, 1-396 Kosugi-cho, Nakahara-ku, Kawasaki, Kanagawa 211-8533, Japan

## Abstract

The physical properties of the extracellular matrix (ECM), such as stiffness, are involved in the determination of the characteristics of cancer cells, including chemotherapy sensitivity. Resistance to chemotherapy is often linked to dysfunction of tumor suppressor p53; however, it remains elusive whether the ECM microenvironment interferes with p53 activation in cancer cells. Here, we show that, in MCF-7 breast cancer cells, extracellular stiffness influences p53 activation induced by the antitumor drug doxorubicin. Cell growth inhibition by doxorubicin was increased in response to ECM rigidity in a p53-dependent manner. The expression of Rho-associated coiled coil-containing protein kinase (ROCK) 2, which induces the activation of myosin II, was significantly higher when cells were cultured on stiffer ECM substrates. Knockdown of ROCK2 expression or pharmacological inhibition of ROCK decreased doxorubicin-induced p53 activation. Our results suggest that a soft ECM causes downregulation of ROCK2 expression, which drives resistance to chemotherapy by repressing p53 activation.

## 1. Introduction

One of the major causes of resistance to cancer therapies, such as chemotherapy and radiation, is dysfunction of p53 [1–3]. Under low stress conditions, the expression of p53 is maintained at a low level due to Mdm-2-mediated degradation [4]. In response to DNA damage caused by genotoxic drugs, PI3 kinase-like kinase family members (ATM, ATR, and DNA-PK) are activated and then phosphorylate p53 [5, 6]. Subsequently, they phosphorylate and stabilize p53 by attenuating its dissociation from Mdm2-mediated degradation [7]. p53 then induces cell cycle arrest and apoptosis by inducing the expression of various target genes, including* p21*^*WAF1*^,* NOXA*, and* PIG6*, leading to growth inhibition of damaged cells [8]. p53 dysfunction is frequently caused by mutation(s) in* TP53* encoding p53 [9].

Accumulating evidence has shown that extracellular matrix (ECM) stiffness, a tumor microenvironment factor, contributes to chemotherapy sensitivity [10]. Stiffness of the ECM secreted by stroma cells, such as cancer-associated fibroblasts and myofibroblasts, is increased in most solid tumors [11, 12]. For example, mammary gland tumors, whose elastic modulus is approximately 1–4 kPa, are stiffer than healthy mammary glands (<1 kPa). Notably, stiffer tumors are associated with a higher aggressiveness of cancer cells [11, 13, 14]. However, it remains elusive whether ECM stiffness modulates p53 function and chemotherapy sensitivity in cancer cells.

Cells sense ECM stiffness at integrin-mediated cell-ECM adhesion structures termed focal adhesions (FAs) [15]. The rigidity of the ECM modulates the recruitment and phosphorylation of FA proteins, leading to actin polymerization via the activation of Rho GTPases, including Rho, Rac, and Cdc42 [16]. Rho also activates ROCK, which induces the activation of the actin-based motor protein myosin and increases the generation of actin-myosin contractile forces [11].

Treatment with the genotoxic drug doxorubicin typically induces remodeling of actin cytoskeleton architecture; however, its effects appear somewhat controversial [17–21]. In mouse embryonic fibroblasts, doxorubicin impairs the formation of contractile actomyosin bundles, that is, stress fibers, but induces the formation of cortical actomyosin rings [19, 20]. Conversely, other studies have reported that the formation of stress fibers is promoted by doxorubicin prior to the onset of apoptosis in several cell lines, including MCF-7 cells [17, 21]. While the formation of stress fibers as well as FAs is impaired in suspended cells, the efficacy of doxorubicin to induce apoptosis is increased in these cells [17, 18], suggesting that the formation of stress fibers and FAs may help to protect cells from apoptosis. In addition, it has also been reported that, in mouse embryonic fibroblasts, the formation of filopodia, which are thin finger-like cell protrusions generated by actin polymerization, is diminished upon doxorubicin treatment [22]. Since filopodia protrusions promote the survival of disseminated carcinoma cells [23], the doxorubicin-induced attenuation of filopodia formation may contribute to the low viability of doxorubicin-treated cells.

In this study, we examined whether matrix stiffness affects the doxorubicin-induced growth inhibition of MCF-7 breast cancer cells expressing wild-type p53. We found that doxorubicin treatment reduced cell viability to a larger extent on a stiff substrate (30 kPa) than on a soft one (2 kPa) and that this stiffness-dependent decrease in the viability of doxorubicin-treated cells required p53 activation. ROCK2 expression was increased in response to ECM rigidity, and ROCK2 knockdown diminished doxorubicin-induced p53 activation. The ectopic expression of p53 prevented doxorubicin-induced filopodia formation in cells cultured on soft substrates. Our findings suggest that the upregulation of ROCK2-mediated actomyosin contractility on a stiff ECM confers the chemotherapy response of cancer cells via the substantial activation of p53.

## 2. Materials and Methods

### 2.1. Cell Culture

MCF-7 human breast cancer cells and 293T human embryonic kidney cells obtained from the American Type Culture Collection were cultured in Dulbecco's modified Eagle's medium (Nissui Pharmaceutical) supplemented with 10% fetal bovine serum and 1% penicillin/streptomycin. N-Acryloyl-6-aminocaproic acid- (ACA-) copolymerized acrylamide gels for polyacrylamide culture substrates were prepared as described previously [24, 25]. The cells were seeded on the substrates at 2.2 × 10^5^ cells/cm^2^ and treated with doxorubicin (DOXO; 1 *μ*g/mL) after incubation for 24 h.

### 2.2. MTT Assay

Cell growth was assessed by an MTT assay according to the manufacturer's instructions (Dojindo, Inc.).

### 2.3. Retroviral Vectors and Retroviral Infection

To generate retroviruses encoding small hairpin RNAs (shRNAs) against human* p53* and human* ROCK2*, the* p53* target sequence [26] 5′-GACTCCAGTGGTAATCTAC-3′ and* ROCK2* target sequence [27] 5′-GGTTTATGCTATGAAGCTT-3′ were cloned into the pSuper retro puro vector (Oligoengine). Retroviral infection was performed as described previously [28]. Briefly, the retrovirus vector encoding shRNA was cotransfected with the pAmpho plasmid into 293T cells using the HilyMax transfection reagent (Dojindo). At 48 h after transfection, the supernatant was collected and then used to infect MCF-7 cells in the presence of 8 *μ*g/mL Polybrene. Infected cells were selected using puromycin (1.5 *μ*g/mL) for 3 days.

### 2.4. Antibodies and Materials

Anti-p53 mouse monoclonal (DO-1; Santa Cruz Biotechnology), anti-HDAC1 mouse monoclonal (2E10; Merck Millipore), anti-*α*-tubulin mouse monoclonal (DM1A; Sigma-Aldrich), anti-p21 rabbit monoclonal (EPR3993; Abcam), and anti-Lamin B1 rabbit polyclonal (Abcam) antibodies were used for immunoblot analyses. Anti-pMLC2 (Ser19) rabbit polyclonal (Cell Signaling Technology) and anti-HA mouse monoclonal (16B12; Covance) antibodies were used for immunofluorescence analyses. Doxorubicin and Y-27632 were purchased from Merck Millipore.

### 2.5. Quantitative Real-Time PCR

Quantitative real-time PCR analysis was performed as described previously [29]. The following primers were used: human* p21*^*waf1*^ forward 5′-GGCTTCATGCCAGCTACTTC-3′ and reverse 5′-CCCTAGGCTGTGCTCACTTC-3′; human* NOXA* forward 5′-AGCTGGAAGTCGAGTGTGCT-3′ and reverse 5′-ACGTGCACCTCCTGAGAAAA-3′; human* PIG6* forward 5′-TTTTTCACCCCACACTTGCAGA-3′ and reverse 5′-TGTCCCAGGCAGGTATCAGGTT-3′; human* ROCK2* forward 5′-CAACTGTGAGGCTTGTATGAAG-3′ and reverse 5′-TGCAAGGTGCTATAATCTCCTC-3′; and human* ubiquitin* forward 5′-TGACTACAACATCCAGAA-3′ and reverse 5′-ATCTTTGCCTTGACATTC-3′ [24].

### 2.6. Immunoblot Analysis

MCF-7 cells were solubilized with the lysis buffer (50 mM Tris pH 7.4, 150 mM NaCl, 1% Triton X-100, 1% SDS, 10 mM EDTA, 1 mM Na_3_VO_4_, 10 mM NaF, and protease inhibitor cocktail [PIC; Nacalai Tesque]) and then centrifuged at 20,000 ×g for 20 min after sonication. The supernatants were used as total cell extracts and subjected to sodium dodecyl sulfate-polyacrylamide gel electrophoresis (SDS-PAGE). For fractionation of nuclear and cytosol extracts, the cells were solubilized in buffer A (10 mM HEPES pH 7.2, 10 mM KCl, 0.1 mM EDTA, 0.1 mM EGTA, 0.4% NP-40, and PIC) and then centrifuged at 10,000 ×g for 10 min after incubation on ice for 5 min. The supernatants were used as cytosol extracts. The pellets were washed with buffer A and subsequently resuspended in buffer B (20 mM HEPES pH 7.9, 400 mM NaCl, 1 mM EDTA, 1 mM EGTA, and PIC) in a vortex mixer for 10 min. The supernatants obtained after centrifugation at 20,000 ×g for 15 min were used as nuclear extracts. The extracts were subjected to SDS-PAGE. To obtain the Triton X-100 soluble and insoluble fractionations, the cells were solubilized with the lysis buffer (50 mM Tris pH 7.4, 150 mM NaCl, 1% Triton X-100, 10 mM EDTA, 1 mM Na_3_VO_4_, 10 mM NaF, and PIC) and then at 20,000 ×g for 15 min after incubation on ice for 15 min. The supernatants were used as Triton X soluble fractions. The pellets were resuspended in the SDS sample buffer after washing with the buffer and subsequently sonicated. The lysates were used as Triton X insoluble fractions. The extracts were subjected to SDS-PAGE.

### 2.7. Fluorescence Microscopy

To immunostain for MLC2 phosphorylated at Ser19, the cells were fixed with 10% formaldehyde in phosphate-buffered saline (PBS) supplemented with 0.1 M HEPES pH 7.4 and then permeabilized with 0.2% Triton X-100. After blocking with 5% goat serum in PBS, the cells were incubated with the anti-pMLC2 (Ser19) antibody. Alexa Fluor 633-conjugated goat anti-rabbit IgG (Molecular Probes) was used as a secondary antibody. Alexa Fluor 488 phalloidin (Molecular Probes) and DAPI (Vector Laboratories) were used to stain F-actin and nuclei, respectively. To visualize p53 and F-actin simultaneously in single cells within spheroids, cells cultured overnight on the 2 kPa substrate were transfected with the Lifeact-GFP expression vector (a gift from Roland Wedlich-Söldner, University of Munster, Munster, Germany [30]) together with the control or HA-tagged p53 expression vector. After 24 h, the cells were treated with doxorubicin for 16 h. The cells were fixed with 10% formaldehyde in PBS containing 0.1 M HEPES pH 7.4. Doxorubicin incorporated into the cells was visualized using its autofluorescence (e.g., ex: 488 nm/em: >580 nm [31]). Images were acquired using a confocal microscope (LSM700; Zeiss) and then analyzed with ImageJ software (NIH).

### 2.8. Statistical Analysis

Statistical analysis of data was performed using the unpaired Student's two-sided *t*-test.

## 3. Results and Discussion

**Figure 1:**
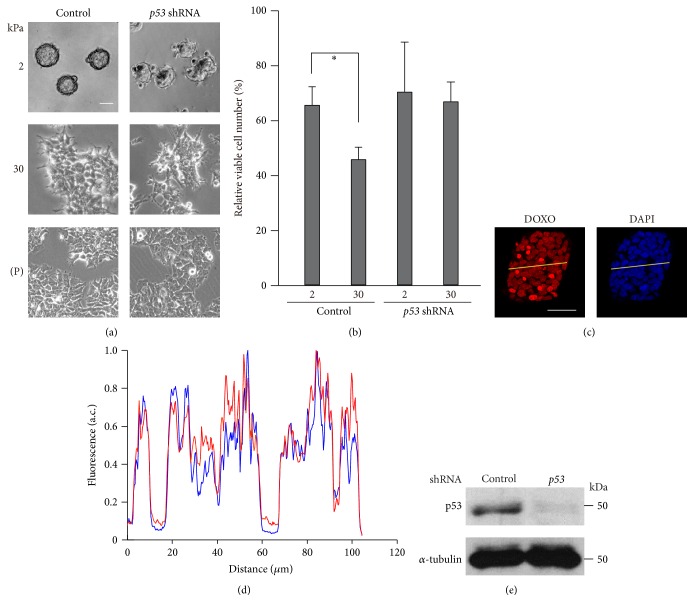
Rigid substrates enhance the inhibitory effect of doxorubicin on cell growth in a p53-dependent manner. (a-b) MCF-7 cells infected with a control or* p53* shRNA-expressing retrovirus were cultured on substrates with elasticities of 2 and 30 kPa or on plates (P; ~10^6^ kPa). (a) Phase contrast images of the cells were obtained with an inverted microscope (Olympus CKX41). Scale bar, 50 *μ*m. (b) The cells were treated with doxorubicin (DOXO; 1 *μ*g/mL) for 24 h, and the number of viable cells was counted using trypan blue exclusion-based cell staining. In each condition, the number of doxorubicin-treated cells was normalized to that of nontreated cells. Each bar represents the mean ± standard deviation (SD); *n* = 4. Asterisks, *p* < 0.05. (c-d) Accumulation of doxorubicin into cells cultured on a substrate with an elasticity of 2 kPa was evaluated. (c) Doxorubicin incorporated into cells was visualized using its autofluorescence. Z-stack images with an interval of 1.0 *μ*m were obtained using a confocal microscope. Projected images for doxorubicin (red) and nuclei (DAPI) are shown. Scale bar, 50 *μ*m. (d) The fluorescence intensity of the yellow line drawn across the spheroid was plotted. Intensity values were normalized with respect to the maximum value in each profile. (e) Extracts from control and* p53* shRNA-expressing cells cultured on plates were subjected to immunoblot analysis with antibodies against p53 and *α*-tubulin as a loading control.

We cultured MCF-7 cells on gelatin-coated ACA gels with elasticities of 2 kPa and 30 kPa as well as on plastic dishes (elastic modulus ~10^6^ kPa). The cells on the 2 kPa substrate aggregated and formed spheroid structures (Figure 1(a)). Conversely, the cells cultured on the 30 kPa substrate exhibited flat and spread morphologies, although they were flatter and more spread out when cultured on plastic dishes. While activation of integrin signaling in response to a rigid ECM can prevent antitumor drug-induced cell death [32], spheroid formation, which is typically observed on a soft ECM (Figure 1(a)), also reportedly makes cancer cells resistant to antitumor drugs [33, 34]. Given these apparently opposing results, we examined how the inhibitory effect of doxorubicin on cell growth was influenced by ECM stiffness using trypan blue exclusion-based cell staining. When the cells were cultured on the 2 kPa substrate, doxorubicin treatment reduced the relative number of viable cells by ~35% (Figure 1(b)). Conversely, when the cells were cultured on the 30 kPa substrate, the relative number of viable cells was reduced further (by ~54%) upon doxorubicin treatment. We further investigated the effect of ECM stiffness on cell proliferation in the presence or absence of doxorubicin using an MTT assay. The number of cells cultured on the 30 kPa substrate was significantly more than that cultured on the 2 kPa substrate (see Figure S1 in Supplementary Material available online at https://doi.org/10.1155/2017/5158961), indicating that substrate stiffening promotes cell proliferation. By contrast, the number of doxorubicin-treated cells cultured on the 30 kPa substrate was similar to that cultured on the 2 kPa substrate. The doxorubicin-induced attenuation of cell proliferation was also more significant on the 30 kPa substrate than on the 2 kPa substrate.

While MCF-7 cells cultured on the soft substrate form spheroids (Figure 1(a)), spheroid formation provides hypoxic microenvironments and attenuates drug penetration [33, 34]. These effects associated with spheroid formation potentially contribute to the chemotherapeutic resistance of cancer cells, in which downregulation of p53 activation is reportedly involved [33]. However, the following results suggest that downregulation of p53 activation on the soft substrate is unlikely to be caused by spheroid-associated hypoxia or limited drug penetration in our system. First, although hypoxia typically occurs in tissue located at a distance of 100–150 *μ*m from blood vessels [35], the width of spheroids formed in our experimental condition was much smaller (40 ± 10 *μ*m in radius, *n* = 214). Second, the concentration of penetrated doxorubicin was not apparently different between the central region and outer edge of the spheroids (Figures 1(c) and 1(d)).

We next investigated the contribution of p53 to the ECM stiffness-dependent regulation of cellular morphology and doxorubicin-induced cell growth inhibition. MCF-7 cells with shRNA-mediated depletion of p53 expression (Figure 1(e)) were spread out on the 30 kPa and plastic substrates but formed spheroids on the 2 kPa substrate, albeit these spheroids had irregular shapes compared with those of control cells (Figure 1(a)). This suggests that the p53-depleted cells retain the ability to sense differences in substrate elasticity. However, the rigidity-dependency of the doxorubicin-induced reduction of cell viability (Figure 1(b)) and cell proliferation (Fig. S1) was abrogated upon p53 knockdown.

**Figure 2:**
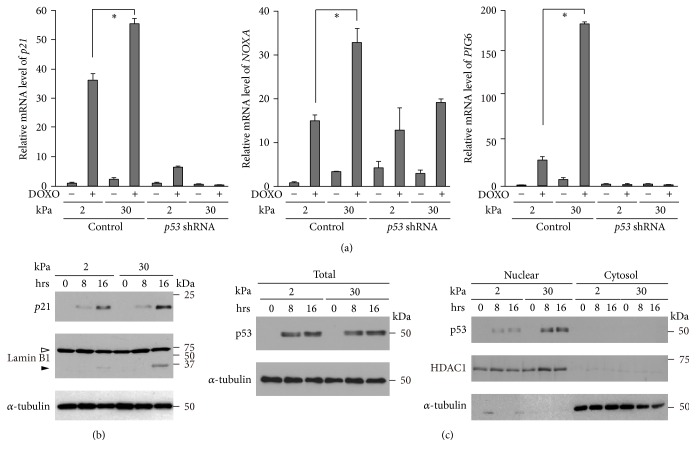
Soft substrates diminish doxorubicin-induced p53 activation. (a–c) Cells infected with a control or* p53* shRNA-expressing retrovirus were cultured on substrates with elasticities of 2 and 30 kPa. (a) The expression of* p21*^*Waf1*^,* NOXA,* and* PIG6* in cells cultured in the presence or absence of DOXO (1 *μ*g/mL) for 24 h was evaluated by quantitative real-time PCR. Each bar represents the mean ± SD; *n* = 3. Asterisks, *p* < 0.01. (b-c) Control and* p53* shRNA-expressing cells were treated with DOXO for the indicated time periods. (b) Total cell lysates were subjected to immunoblot analysis with antibodies against p21, Lamin B1, and *α*-tubulin as a loading control. The white arrowhead indicates full-length Lamin B1, while the black arrowhead indicates a cleaved fragment. (c) Total cell extracts, nuclear extracts, and cytosol extracts were subjected to immunoblot analysis with antibodies against p53, HDAC1 as a nuclear marker, and *α*-tubulin as a loading control or cytosol marker.

To dissect whether p53 activation was involved in the ECM rigidity-dependent modulation of doxorubicin-induced cell growth inhibition, we examined p53 transcriptional activity by evaluating the expression of its well-known target genes,* p21*^*Waf1*^, which encodes a cyclin-dependent kinase inhibitor, and* NOXA* and* PIG6*, whose products induce apoptosis by activating caspases [8]. The expression of* p21*^*Waf1*^ was drastically increased by doxorubicin treatment in a p53-dependent manner, and its doxorubicin-induced expression was significantly higher on the 30 kPa substrate than on the 2 kPa substrate (Figures 2(a) and 2(b)). We observed similar effects of substrate stiffness and p53 on the gene expression of* NOXA* and* PIG6*. Consistent with the increased gene expression of these apoptosis-inducing factors (Figure 2(a)), cleavage of the caspase substrate Lamin B1 [36–40] was also increased by doxorubicin treatment in a substrate rigidity-dependent manner (Figure 2(b)). Thus, a stiff ECM has a promoting effect on doxorubicin-induced p53 activation, which would underlie the observation that cell growth inhibition following doxorubicin treatment is enhanced on a stiff ECM in a p53-dependent manner (Figure 1(b)).

We next examined whether p53 protein levels following doxorubicin treatment were influenced by ECM stiffness. The expression of p53 increased upon doxorubicin treatment, which was not affected by substrate rigidity (Figure 2(c) left). Conversely, the amount of p53 in the nuclear fraction was larger when the cells were cultured on the stiffer substrate (Figure 2(c) right). By contrast, the amount of p53 in the Triton X-100 insoluble cytoskeleton fraction [41] of doxorubicin-treated cells was lower on the 30 kPa substrate than on the 2 kPa substrate (Fig. S2). These results suggest that a soft ECM attenuates the nuclear accumulation of p53 through the interaction of p53 with the cytoskeleton.

**Figure 3:**
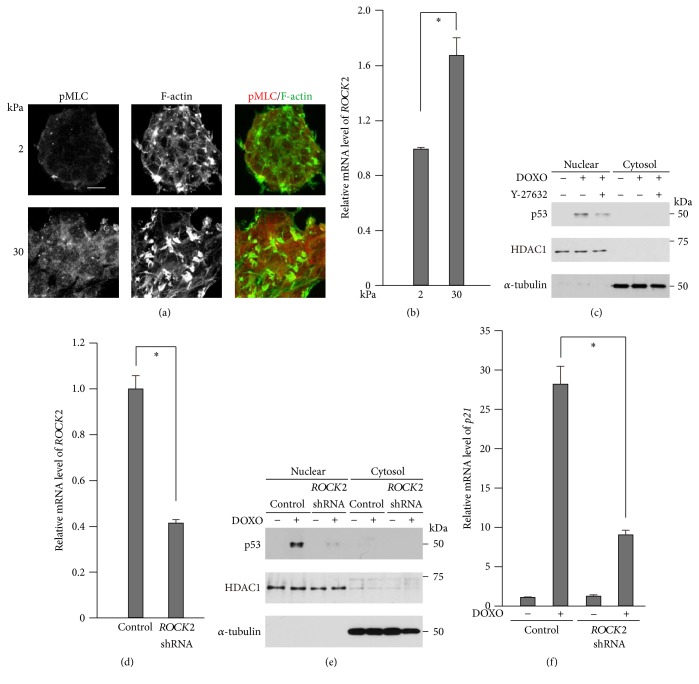
ROCK2 is involved in doxorubicin-induced p53 activation. (a) Cells were cultured on substrates with elasticities of 2 and 30 kPa. Confocal images of cells stained for phosphorylated MLC (pMLC2; red) and F-actin (green). Scale bars, 20 *μ*m. Z-stack images with an interval of 1.0 *μ*m were obtained using a confocal microscope, and projected images are shown. (b)* ROCK2* expression in cells cultured on substrates with elasticities of 2 and 30 kPa was evaluated by quantitative real-time PCR. Each bar represents the mean ± SD; *n* = 3. Asterisks, *p* < 0.02. (c) Nuclear extracts and cytosol extracts from cells cultured in the presence or absence of DOXO (1 *μ*g/mL) and/or Y-27632 (10 *μ*M) for 16 h were subjected to immunoblot analysis with antibodies against p53, HDAC1 as a nuclear marker, and *α*-tubulin as a cytosol marker. (d–f) The cells were infected with a control or* ROCK2* shRNA-expressing retrovirus. (d)* ROCK2* expression was evaluated as in (b). (e) Nuclear extracts and cytosol extracts from cells cultured in the presence or absence of DOXO for 16 h were subjected to immunoblot analysis with antibodies against with p53, HDAC1 as a nuclear marker, and *α*-tubulin as a loading control or cytosol marker. (f)* p21*^*Waf1*^ expression in cells cultured in the presence or absence of DOXO for 24 h was evaluated by quantitative real-time PCR. Each bar represents the mean ± SD; *n* = 3. Asterisks, *p* < 0.01.

Stiff substrates have been shown to increase actomyosin contraction [11, 42, 43]. We have also shown that the expression of the actomyosin activator ROCK2 is downregulated in fibroblasts when the cells are cultured on soft (<4 kPa) substrates [44]. Consistent with these previous reports, phosphorylation of myosin light chain (MLC), a critical step in the activation of nonmuscle myosin, was higher in cells cultured on stiffer substrates (Figure 3(a)). Since it has been reported that the doxorubicin-induced stabilization and concomitant nuclear accumulation of p53 are suppressed by the myosin II ATPase inhibitor blebbistatin or the ROCK inhibitor Y-27632 in keratinocytes [45], we asked whether rigidity-dependent ROCK2 expression and concomitant actomyosin activation were involved in the enhanced activation of p53 on the stiff substrate. Consistent with the case of fibroblasts [44],* ROCK2* expression in MCF-7 cells was significantly higher on the 30 kPa substrate than on the 2 kPa substrate (Figure 3(b)). While doxorubicin treatment induced the accumulation of p53 in nuclei (Figure 2(b)), both ROCK inhibition (Figure 3(c)) and ROCK2 knockdown (Figures 3(d) and 3(e)) diminished the doxorubicin-induced nuclear accumulation of p53. Concomitantly, the expression of the p53 target gene* p21*^*Waf1*^, which was induced upon doxorubicin treatment, was largely decreased by ROCK2 knockdown (Figure 3(f)). Taken together, these results suggest that the substrate stiffness-dependent increase in the expression and activity of ROCK2 contributes to p53 activation.

The ROCK family consists of ROCK1 and ROCK2. Both isoforms have redundant functions and are implicated in MLC2 phosphorylation upon doxorubicin treatment [19, 20]. While we have shown here that ROCK2 activity potentiates the doxorubicin-induced activation of p53, previous studies have suggested that p53 activation can, in turn, lead to the enhanced activation of ROCK1. While ROCK1 is constitutively activated during apoptosis upon its cleavage by caspase-3 and caspase-7 [46], p53 activates these caspases [47]. Therefore, ROCKs and p53 potentially form a positive feedback loop in cells undergoing apoptosis. This positive feedback mechanism would accelerate cell depletion upon doxorubicin treatment, and ECM rigidity may affect the efficacy of doxorubicin on cell depletion by modulating this feedback mechanism. However, the actual mechanism by which ROCK regulates p53 activity remains unclear. Since p53 shuttles dynamically between the nucleus and cytoplasm [48], ROCK may modulate the import/export of p53 into/from the nucleus to regulate the nuclear localization of p53, which needs to be examined in future studies.

**Figure 4:**
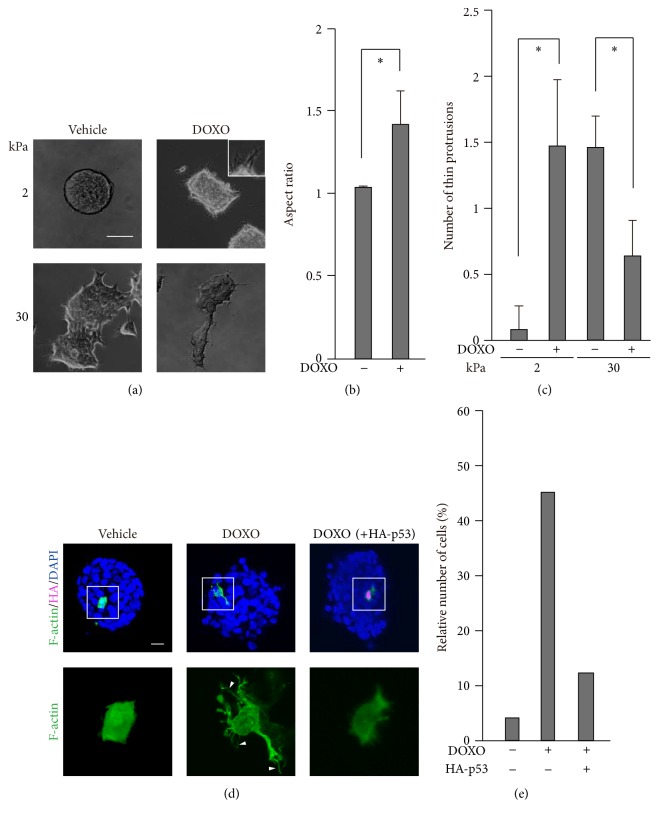
Doxorubicin treatment induces protrusion formation in cells cultured on soft substrates. (a, d) Cells cultured on substrates with elasticities of 2 kPa (a, d) and 30 kPa (d) were treated with or without DOXO (1 *μ*g/mL) for 16 h. (a) Phase contrast images of the cells were obtained with an inverted microscope. Scale bar, 100 *μ*m. (b) The aspect ratio of spheroids cultured on 2 kPa was calculated from the major length to the minor length of an ellipse that fitted each spheroid using ImageJ software. Each bar represents the mean ± SD; *n* = 10. Asterisks, *p* < 0.01. (c) The number of thin protrusions (≧20 *μ*m) along the periphery of a cell cluster was quantified using ImageJ software. Data indicate the number of protrusions per 100 *μ*m of the periphery of a cell cluster. Each bar represents the mean ± SD; *n* = 5. Asterisks, *p* < 0.01. (d) The cells were transfected with the Lifeact-GFP expression vector to label F-actin together with the HA-tagged p53 expression vector before treatment with DOXO. Confocal images of F-actin (green), HA (magenta), and nuclei (DAPI; blue) are shown. Z-stack images with an interval of 1.0 *μ*m were obtained using a confocal microscope, and projected images are shown. Magnified images of F-actin in the boxed regions are also shown. Scale bar, 20 *μ*m. The white arrowheads indicate filopodia-like protrusions (≧10 *μ*m). (e) The relative number of cells, which have more than three filopodia-like protrusions (≧10 *μ*m), to total cell number (*n* = 24), is shown.

p53 alters cell behavior through remodeling of the actin cytoskeleton [49], and actin remodeling is involved in the determination of cell fate upon doxorubicin treatment [17, 19–22, 50]. While actin remodeling plays a central role in the morphological changes of cells [51], we noticed that the spheroids that formed on the 2 kPa substrate spread out with thin protrusions after treatment with doxorubicin (Figures 4(a)–4(c)). By contrast, cell clusters on the 30 kPa substrate became less spread out upon doxorubicin treatment, which accompanied a decrease in the formation of thin protrusions (Figures 4(a) and 4(c)). We then examined how doxorubicin modulated the actin cytoskeleton to induce protrusion formation in cells cultured on the 2 kPa substrate. Doxorubicin treatment caused the formation of long filopodia-like protrusions in cells residing in spheroids (Figures 4(d) and 4(e)). Importantly, while p53 activity was relatively low on the 2 kPa substrate compared with the stiffer substrate (Figure 2), the ectopic expression of p53 in cells on the 2 kPa substrate abrogated the doxorubicin-induced formation of protrusions (Figures 4(d) and 4(e)). This inhibitory effect of p53 on protrusion formation is consistent with the previously reported functions of p53; p53 decreases the activity of Cdc42, a small GTPase that promotes the formation of filopodia, as well as the expression of fascin, a major actin-bundling protein in filopodia [22, 52, 53]. Considering that filopodia protrusions promote cell survival [23], our results imply that the reduced activity of p53 in spheroids may make cells resistant to chemotherapy by promoting protrusion formation.

The stemness of cancer cells is a contributing factor to the metastasis, recurrence, and chemotherapy resistance of cancers [54–57]. p53 reduces stemness by repressing the expression of various stem cell markers and by activating the DNA excision repair pathway [49, 58]. Conversely, ROCK inhibition and soft substrates promote the self-renewal of stem cells and reprogramming of fibroblasts into stem cells [50–54]. Given these previous results, downregulation of the ROCK-p53 axis on soft substrates, which we have revealed in this study, may contribute to the production of cancer stem cells.

## 4. Conclusions

Our study provides novel insights into the mechanism underlying the environment-mediated drug resistance of breast cancer cells. Stiffer substrates make breast cancer cells more susceptible to doxorubicin treatment in a p53-dependent manner. It is of note that while advanced cancer cells are associated with stiffer tumors [9, 11, 12], they typically bear somatic mutations of* TP53* at a high rate [9]. Therefore, due to p53 dysfunction by its mutation, advanced cancer cells may exhibit chemotherapeutic resistance even in rigid tumors. Conversely, cancer cells at early stages, in which the* TP53* gene does not typically suffer severe mutations, reside in relatively soft environments that may reduce the inhibitory effect of genotoxic drugs on cell growth. Treatment of early stage cancer cells with genotoxic drugs combined with a drug or physical method that increases extracellular stiffness and/or myosin II activity may provide an effective approach for cancer therapy.

## Supplementary Material

Fig. S1. Doxorubicin decreases the viability of cells cultured on substrates with an elasticity of 30 kPa compared to those with an elasticity of 2 kPa in a p53-dependent manner. Fig. S2. Soft substrates increase the amount of p53 in the Triton-X insoluble fraction.
