# Supplementary material for: Substrate Stiffness Influences Doxorubicin-Induced p53 Activation via ROCK2 Expression
Source: Biomed Res Int. 2017 Jan 16;2017:5158961. doi: 10.1155/2017/5158961 (PMC5278210; doi:10.1155/2017/5158961)

Supplementary figure legends

Fig. S1. Doxorubicin decreases the viability of cells cultured on substrates with an elasticity of 30 kPa compared to those with an elasticity of 2 kPa in a p53-dependent manner.

MCF-7 cells infected with a control or *p53* shRNA-expressing retrovirus were cultured on substrates with elasticities of 2 and 30 kPa. After incubation for 24 h, the cells were treated with doxorubicin (DOXO; 1 μg/mL) for 24 h. The number of viable cells after seeding was quantified using an MTT assay. Filled circles with a solid line, untreated cells cultured on the 2 kPa substrate; open circles with a broken line, DOXO-treated cells cultured on the 2 kPa substrate; filled triangles with a solid line, untreated cells cultured on the 30 kPa substrate; and open triangles with a broken line: DOXO-treated cells cultured on the 30 kPa substrate. Each bar represents the mean ± standard deviation; n = 3.

Fig. S2. Soft substrates increase the amount of p53 in the Triton-X insoluble fraction.

The Triton-X soluble (S) and insoluble (I) fractions from cells cultured in the presence of DOXO (1 μg/mL) for 16 h on substrates with elasticities of 2 kPa and 30 kPa were subjected to immunoblot analysis with antibodies against p53. Blots of p53 were

quantified and the relative values of the p53 insoluble fraction to the soluble fraction are shown.

Figure S1

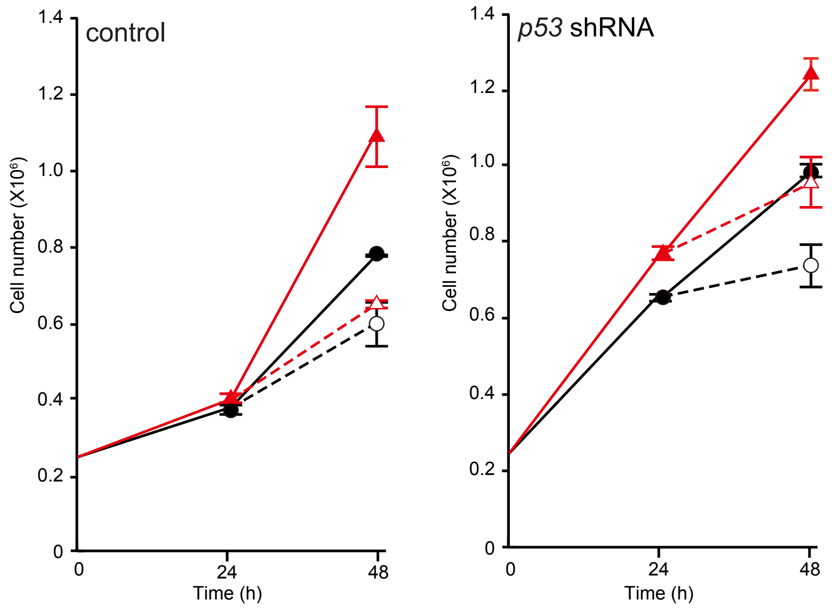

control
Cell number (X10^6)
1.4
1.2
1.0
0.8
0.6
0.4
0.2
0
0
24
48
Time (h)
p53 shRNA
Cell number (X10^6)
Time (h)

Figure S2

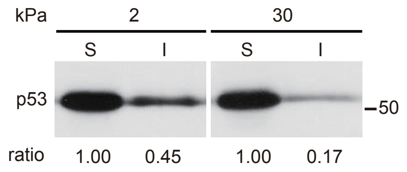

Supplement: Supplementary file 1 — Fig. S1. Doxorubicin decreases the viability of cells cultured on substrates with an elasticity of 30 kPa compared to those with an elasticity of 2 kPa in a p53-dependent manner. Fig. S2. Soft substrates increase the amount of p53 in the Triton-X insoluble fraction. [file 5158961.f1.pdf]
